# 910. Comparable Outcomes, Unequal Access in Pediatric LEP Burn Patients

**DOI:** 10.1093/jbcr/irag033.414

**Published:** 2026-04-06

**Authors:** Collin Montgomery, Bailey Garnica, Shriya Kane, Colette Galet, Samuel W Jones

**Affiliations:** University of Iowa, Iowa City, IA; University of Iowa Carver College of Medicine, Iowa City, IA; University of Iowa, Iowa City, IA; University of Iowa, Iowa City, IA; University of Iowa Health Care, Iowa City, IA

## Abstract

**Introduction:**

Pediatric burn care requires complex inpatient management, close outpatient follow-up, and multidisciplinary care coordination for recovery, wound care, and rehabilitation. Access to resources such as primary care providers (PCPs), insurance coverage, rehabilitation therapy, and outpatient follow-up are critical in this patient population. Language barriers are a well-recognized contributor to pediatric health disparities. Pediatric patients and their families with limited English proficiency (LEP) are more likely to present with increased disease severity, experience delays in care, longer hospital length of stay (LOS), higher rates of postoperative complications, and differences in outpatient follow-up attendance. Herein, we aimed to identify differences in complication rates, LOS, social resources and support, utilization of healthcare resources, and follow-up between pediatric burn patients with LEP compared to English speaking patients.

**Methods:**

This is a retrospective cohort study of pediatric burn admissions from 2015 to 2025 at an ABA certified burn center. LEP patients were propensity matched 2:1 to non-LEP patients based on age, sex, and total burn surface area (TBSA). Data collected included demographics, inpatient social work assessments, inpatient rehabilitation therapy, surgeries performed, complications, delays in discharge, outpatient services, outpatient follow-up, and unplanned readmissions. Categorical variables were compared using Chi-square or Fisher exact tests and continuous variables using Mann–Whitney U tests. *p* value <0.05 was considered statistically significant.

**Results:**

A total of 57 LEP patients were included and propensity matched to 109 non-LEP patients. LEP patients were more likely to be transferred from another facility (94.9% vs. 84.1 %, *p*=.047) and to undergo in-hospital rehabilitation therapy (71.2% vs. 43.9%, *p*=.001). There was no difference in complication rates (6.8% vs. 9.3%, *p*=.772), undergoing evaluation by social work (77.6% vs. 82.8%, *p*=.529), LOS (1 [1-4] vs. 1 [1-4], *p*=.868), delays in discharge (13.6% vs. 10.3%, *p*=.612), or being lost to follow-up (25.4% vs. 25.7%, *p*>.999). However, LEP patients evaluated by social work were less likely to have insurance (80% vs. 96.3%, *p*=.008) and less likely to have a PCP (72.1% vs. 93.8%, *p*=.002).

**Conclusions:**

LEP pediatric burn patients had comparable inpatient outcomes to non LEP patients but had significantly less pre-existing access to care, lacking insurance and PCP. Higher utilization of inpatient rehabilitation therapy may demonstrate anticipated outpatient barriers requiring adjusted inpatient resource allocation.

**Applicability of Research to Practice:**

These findings highlight opportunities for burn centers to address structural barriers by embedding insurance navigation and primary care linkage into standard care pathways for LEP families.

**Funding for the study:**

N/A.

